# Associations of dynamic driving pressure and mechanical power with postoperative pulmonary complications–posthoc analysis of two randomised clinical trials in open abdominal surgery

**DOI:** 10.1016/j.eclinm.2022.101397

**Published:** 2022-04-16

**Authors:** Michiel T.U. Schuijt, Liselotte Hol, Sunny G. Nijbroek, Sanchit Ahuja, David van Meenen, Guido Mazzinari, Sabrine Hemmes, Thomas Bluth, Lorenzo Ball, Marcelo Gama–de Abreu, Paolo Pelosi, Marcus J. Schultz, Ary Serpa Neto

**Affiliations:** a Department of Intensive Care, Amsterdam UMC, location AMC, Amsterdam, The Netherlands; b Department of Anaesthesiology, Amsterdam UMC, location AMC, Amsterdam, The Netherlands; c Department of Anaesthesiology, Pain Management & Perioperative Medicine, & Outcomes Research Consortium Cleveland Clinic, Henry Ford Health System, Detroit, Michigan, The United States of America; d Department of Anaesthesiology, Hospital Universitario y Politécnico la Fe, Valencia, Spain; e Department of Anaesthesiology and Critical Care Medicine, Pulmonary Engineering Group, University Hospital Carl Gustav Carus, Technische Universität Dresden, Dresden, Germany; f Department of Surgical Sciences and Integrated Diagnostics, University of Genoa, Genoa, Italy; g Department of Anaesthesia and Critical Care, San Martino Policlinico Hospital – IRCCS for Oncology and Neurosciences, Genoa, Italy; h Department of Intensive Care and Resuscitation, Cleveland Clinic, Cleveland, Ohio, The United States of America; i Department of Outcomes Research, Anaesthesiology Institute, Cleveland Clinic, Cleveland, Ohio, The United States of America; j Mahidol Oxford Tropical Medicine Research Unit (MORU), Mahidol University, Bangkok, Thailand; k Nuffield Department of Medicine, University of Oxford, Oxford, The United Kingdom; l Department of Medical Affairs, Hamilton Medical AG, Bonaduz, Switzerland; m Australian and New Zealand Intensive Care Research Centre (ANZIC–RC), Monash University, Melbourne, Australia; n Department of Critical Care, Melbourne Medical School, University of Melbourne, Austin Hospital, Melbourne, Australia; o Department of Critical Care Medicine, Hospital Israelita Albert Einstein, São Paulo, Brazil; p Cardio–Pulmonary Department, Pulmonary Division, Faculdade de Medicina, Instituto do Coração, Hospital das Clinicas HCFMUSP, Universidade de Sao Paulo, Sao Paulo, Brazil

**Keywords:** Mechanical ventilation, Postoperative pulmonary complication, Driving pressure, Mechanical power, Intraoperative, Intensity of ventilation

## Abstract

**Background:**

While an association of the intraoperative driving pressure with postoperative pulmonary complications has been described before, it is uncertain whether the intraoperative mechanical power is associated with postoperative pulmonary complications.

**Methods:**

Posthoc analysis of two international, multicentre randomised clinical trials (ISRCTN70332574 and NCT02148692) conducted between 2011–2013 and 2014–2018, in patients undergoing open abdominal surgery comparing the effect of two different positive end–expiratory pressure (PEEP) levels on postoperative pulmonary complications. Time–weighted average dynamic driving pressure and mechanical power were calculated for individual patients. A multivariable logistic regression model adjusted for confounders was used to assess the independent associations of driving pressure and mechanical power with the occurrence of a composite of postoperative pulmonary complications, the primary endpoint of this posthoc analysis.

**Findings:**

In 1191 patients included, postoperative pulmonary complications occurrence was 35.9%. Median time–weighted average driving pressure and mechanical power were 14·0 [11·0–17·0] cmH_2_O, and 7·6 [5·1–10·0] J/min, respectively. While driving pressure was not independently associated with postoperative pulmonary complications (odds ratio, 1·06 [95% CI 0·88–1·28]; *p*=0.534), the mechanical power had an independent association with the occurrence of postoperative pulmonary complications (odds ratio, 1·28 [95% CI 1·05–1·57]; *p*=0.016). These findings were independent of body mass index or the level of PEEP used, i.e., independent of the randomisation arm.

**Interpretation:**

In this merged cohort of surgery patients, higher intraoperative mechanical power was independently associated with postoperative pulmonary complications. Mechanical power could serve as a summary ventilatory biomarker for the risk for postoperative pulmonary complications in these patients, but our findings need confirmation in other, preferably prospective studies.

**Funding:**

The two original studies were supported by unrestricted grants from the European Society of Anaesthesiology and the Amsterdam University Medical Centers, Location AMC. For this current analysis, no additional funding was requested. The funding sources had neither a role in the design, collection of data, statistical analysis, interpretation of data, writing of the report, nor in the decision to submit the paper for publication.

**Research in context**

**Evidence before this study**

It is uncertain whether mechanical power, a summary parameter for intensity of ventilation that has been shown to have an association with mortality in critically–ill patients, also has an association with occurrence of postoperative pulmonary complications in surgery patients. We conducted a search of MEDLINE, Embase, CINAHL, and Web of Science between 1989 – March 2022 with various search terms for mechanical power and postoperative pulmonary complications. The search identified one posthoc analysis of a randomised clinical trial in 1156 adult patients undergoing major non–cardiothoracic, non–intracranial surgery, conducted in an academic tertiary hospital in Melbourne; that study, comparing a low versus high tidal volume, found that the highest intraoperative mechanical power had an independent association with occurrence of postoperative pulmonary complications.

**Added value of this study**

In this current analysis, using individual intraoperative ventilation data of patients at risk for postoperative pulmonary complications and scheduled for open abdominal surgery that were included in two recent international randomised clinical trials of a low versus a high level of positive end–expiratory pressure, we found that higher time–weighted average mechanical power had an independent association with occurrence of postoperative pulmonary complications. The association was neither affected by the level of positive end–expiratory pressure nor by body mass index.

**Implications of all evidence available**

Exposure to higher mechanical power has an independent association with occurrence of postoperative pulmonary complications in at risk patients undergoing open abdominal surgery. Next to being a useful digital biomarker, with prognostic capacities for outcome, mechanical power may also serve as a target, to limit the intensity of intraoperative ventilation.

Alt-text: Unlabelled box

## Introduction

Postoperative pulmonary complications often occur in patients after major surgery and result in attributable morbidity and mortality.^1^, ^2^, ^3^ Previous work suggests that a high tidal volume,^4^ a high driving pressure,^5^, ^6^, ^7^, ^8^ and also a high flow and a high respiratory rate^4^ can induce lung injury. Changing one of these variables could inadvertently affect the other three variables. For example, lowering of tidal volume, and with that driving pressure, could prompt a higher respiratory rate to avoid hypercapnia due to hypoventilation.

The mechanical power of ventilation is a recently introduced ventilation parameter that summarizes the amount of energy per unit of time transferred from the ventilator to the respiratory system, and part of this energy acts directly on lung tissue where it can cause iatrogenic harm.^9^ The mechanical power can be calculated from tidal volume, respiratory rate, driving pressure, and peak pressure in volume–controlled ventilation.^9^ These individual variables interact, while mechanical power combines multiple variables, both static and dynamic. In critically–ill patients, both driving pressure and the mechanical power have been shown to have associations with morbidity and mortality.^9^, ^10^, ^11^, ^12^, ^13^, ^14^, ^15^, ^16^

The effects of the intraoperative mechanical power on the occurrence of postoperative pulmonary complications in abdominal surgery have not yet been investigated thoroughly. In theory, limiting the intraoperative mechanical power could reduce the risk for postoperative pulmonary complications and as such improve postoperative outcomes. We tested the hypothesis that the mechanical power has an association with occurrence of postoperative pulmonary complications, independent of driving pressure, using individual patient data from two prospective trials that compared postoperative pulmonary complications at different levels of positive end–expiratory pressure (PEEP) in patients at risk for postoperative pulmonary complications planned for open abdominal surgery.

## Methods

### Design, settings and participants

Individual patient data of two recent international, multicentre, randomised clinical trials–the ‘Protective ventilation using high versus low PEEP’ (PROVHILO) trial (ISRCTN70332574)^17^ and the ‘Protective intraoperative ventilation with higher versus lower levels of PEEP in obese patients’ (PROBESE) trial (NCT02148692).^18^ The two studies share several similarities, including patient population (with exclusion of BMI), intervention and control. In addition, both studies used very similar CRFs and datasets. Prior to start of the current analysis, the clinical report forms, data dictionaries and study protocols of the trials were compared, and all definitions in each dataset were discussed among authors to inform the final structure and specification of the combined dataset. Similar variables and parameters were double–checked for consistency across the trials prior to being finally imported into the combined database.

Inclusion and exclusion criteria of the two studies have been reported before;^17^^,^^18^ in short, adult patients with an intermediate or high risk for postoperative pulmonary complications–according to the ‘Assess Respiratory Risk in Surgical Patients in Catalonia’ (ARISCAT) risk score (i.e., with an ARISCAT ≥ 26),^19^^,^^20^ and undergoing general anaesthesia for major abdominal surgery were included. For the current analysis, we excluded patient undergoing laparoscopic abdominal surgery. We also excluded patients with insufficient data to calculate intraoperative driving pressure and the mechanical power.

### Data collection

As dictated by the protocol of the two studies, several baseline parameters, like medical history and vitals, were collected preoperatively. Intraoperative ventilation parameters were manually collected at the start of invasive ventilation and hourly thereafter until the end of surgery. Besides, several non–ventilator parameters, like intraoperative fluid management and prophylactic antibiotic use, were collected.

### Exposure

The driving pressure and the mechanical power were calculated at the start of invasive ventilation, and hourly thereafter until the end of surgery. Since plateau pressure was not recorded in every patient, dynamic driving pressure was calculated as peak pressure – PEEP^21^ and dynamic mechanical power was calculated as 0·098 * tidal volume * respiratory rate * (peak pressure – 0·5 * driving pressure).^9^^,^^22^ To quantify cumulative exposure, time–weighted average driving pressure and mechanical power were calculated as the area under the driving pressure and mechanical power time curve divided by the number of hours of exposure.

### Endpoints

We used the same primary endpoint of the two original studies for this posthoc analysis: postoperative pulmonary complications up to postoperative day seven, defined as a collapsed composite of mild and severe respiratory failure, acute respiratory distress syndrome, pulmonary infection, pleural effusion, atelectasis, pneumothorax, and bronchospasm. Mild and severe respiratory failure were defined as a PaO2 < 60 mmHg or SpO2 < 90% in room air during at least 10 min in need of supplemental oxygen (excluding hypoventilation), and PaO2 < 60 mmHg or SpO2 < 90% despite supplemental oxygen or in need for non–invasive or invasive mechanical ventilation (excluding hypoventilation), respectively. A description of each element of the composite is provided in the Online Supplement.

### Statistical analysis

No sample size calculation was performed, as all available data from the two original trials was used. Patient demographics, ventilation variables, and parameters were presented using descriptive statistics. Continuously distributed variables were expressed as medians and their interquartile ranges. Categorical variables were expressed as n (%). Mann–Whitney U–tests and Chi–squared tests were used to compare groups where appropriate.

To assess the association between the intraoperative mechanical power and postoperative pulmonary complications, a multivariate logistic regression analysis model was used. Covariates were considered for adjustment in all models described based on clinical relevance and were included in the main multivariable logistic regression model by forced entry. The following covariates were considered: age, sex, body mass index, type of surgery, ARISCAT score, American Society of Anesthesiologists (ASA) classification, history of chronic obstructive pulmonary disease, history of active cancer, history of heart failure, preoperative peripheral oxygen saturation, respiratory rate, heart rate, mean arterial blood pressure, fraction of inspired oxygen and end–tidal carbon dioxide in the first hour of intraoperative ventilation, the use of antibiotic prophylaxis, specific procedure, duration of surgery, duration of anaesthesia, use of epidural, type of anaesthesia, amount of blood loss, total amount of fluid administered, use of any transfusion, urine output, emergency procedure and trial. In all models, the participating centre was included as random effect to account for clustering. After checking for collinearity between driving pressure and mechanical power, using gender, BMI, ASA classification score and ARISCAT score as covariates, driving pressure and mechanical power were combined in the same multivariable model with all considered covariates. Missing values were imputed by the median if <5% of data was missing. The E–value was measured, to quantify the evidence strength for the association in the possible presence of unmeasured confounding.^23^ The sample size of the study gives >90% power to detect an effect size (f^2^) as low as 0·05, with 28 or less predictors considering an alpha level of 0·05.

Three sensitivity analyses were performed. The primary analysis was repeated without imputation of missing data. To evaluate the effect of the allocated intervention on the association between the mechanical power and postoperative pulmonary complications, the main model was reassessed for patients in both the low and high PEEP intervention groups. The interaction between PEEP intervention group and intensity of ventilation was assessed. To investigate the effect of body mass index on the association between the mechanical power and postoperative pulmonary complications, the interaction between body mass index and intensity of ventilation was assessed.

All analyses were performed with R statistics (version 3.4.3), and a *p*<0·05 was considered significant.

### Role of the funding source

The two original studies were supported by unrestricted grants from the European Society of Anaesthesiology and the Amsterdam University Medical Centers, Location AMC. For this current analysis, no additional funding was requested. The funding sources had neither a role in the design, collection of data, statistical analysis, interpretation of data, writing of the report, nor in the decision to submit the paper for publication. Schuijt and Serpa Neto had full access to all of the data in the study and takes responsibility for the integrity of the data and the accuracy of the data analysis. All authors took the responsibility to submit the paper for publication.’

## Results

**Figure 1:**
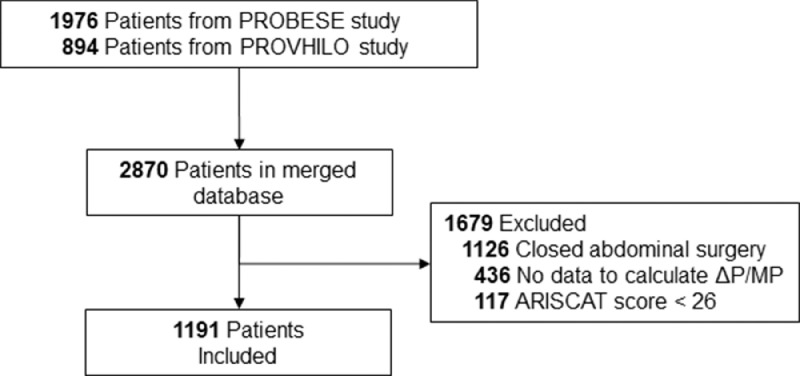
**CONSORT diagram**. PROBESE: Protective Intraoperative Ventilation With Higher Versus Lower Levels of PEEP in Obese Patients; PROBESE: Protective Intraoperative Ventilation With Higher Versus Lower Levels of PEEP in Obese Patients; ΔP: driving pressure; MP: mechanical power. ARISCAT: Assess Respiratory Risk in Surgical Patients in Catalonia

Merging the databases of the two studies resulted in a total of 2870 patients. Of these, 1191 (41·5%) patients could be used for the current posthoc analysis (Figure 1). The main exclusion criteria were laparoscopic abdominal surgery (n = 1126) and insufficient data to calculate driving pressure or the mechanical power (n = 436). The extended CONSORT diagram, including the inclusion of patients per original trial, is presented in eFigures 1 in the Online Supplement. Demographic and surgical characteristics are presented in Table 1. The median age was 65 [57–72] years and 568 (47·7%) patients were men. Most patients had ASA classification score of 2 (48·1%) or 3 (41·1%), and the median ARISCAT score was 41 [34–44]. Following the ARISCAT score, 76·1% of patients had an intermediate and 23·9% of patients had a high risk for developing postoperative pulmonary complications. The most prevalent co–existing disease was active cancer (54·1%). The most common procedure was colorectal surgery (27·0%). The baseline characteristics of the excluded patients because of missing data and of the two original trials are presented in eTable 1 and 2 in the Online Supplement. The main differences were median age (66 versus 56 years), male gender (42·6% versus 61·5%), body mass index (25·5 versus 39·2 kg/m^2^) comparing the PROVHILO and PROBESE study. Besides, the distribution of ASA classification score differed, as 32·9% of patients classified as ASA class 3 in the PROVHILO study, compared to 63·0% of patients in PROBESE study.

**Table 1:** Baseline patient characteristics.

	Participants (n = 1191)
Age, years	65 (57 – 72)
Male gender – no (%)	568 (47·7)
Body mass index, kg/m^2^	27·1 (23·9 – 35·3)
ASA^†^ classification – no (%)	
1	114 (9·6)
2	572 (48·1)
3	489 (41·1)
4	15 (1·3)
ARISCAT score^‡^	41·0 (34·0 – 44·0)
Intermediate – no (%)	906 (76·1)
High – no (%)	285 (23·9)
Risk factors for PPC – no (%)	
SpO_2_	97·0 (96·0 – 98·0)
≥ 96	924 (77·9)
91 – 95	251 (21·2)
< 91	11 (0·9)
Respiratory infection within the last month	66 (5·5)
Anemia*	108 (9·5)
Emergency procedure	40 (3·4)
Coexisiting disorders – no (%)	
Heart failure	196 (16·7)
COPD	92 (7·7)
Active cancer	642 (54·1)
Surgical approach – no (%)	
Non–laparoscopic	1191 (100)
Laparoscopic	0 (0)
Type of surgery – no (%)	
Abdominal	1191 (100)
Non abdominal	0 (0)
Duration of surgery – minutes	190 (138·0 – 276·5)
Specific procedure – no (%)	
Bariatric	24 (2·0)
Bladder or urologic	133 (11·2)
Bowel	32 (2·7)
Colorectal	322 (27·0)
Gastric	94 (7·9)
Gynecology	94 (7·9)
Hepatic	119 (10·0)
Hernia	42 (3·5)
Kidney	25 (2·1)
Pancreatic	128 (10·7)
Vascular	37 (3·1)
Other	141 (11·8)

Data are median (quartile 25% – quartile 75%) or No (%). Percentages may not total 100 because of rounding.

PEEP: positive end expiratory pressure; ARISCAT: Assess Respiratory Risk in Surgical Patients in Catalonia; ASA: American Society of Anesthesiology; PPC: postoperative pulmonary complications.

† The American Society of Anesthesiologists (ASA) criteria for physical status include a classification for normal health (1), mild systemic disease (2), severe systemic disease (3), severe systemic disease that is a constant threat to life (4), and a moribund person who is not expected to survive without the operation (6).

‡ The Assess Respiratory Risk in Surgical Patients in Catalonia (ARISCAT) score estimates the risk of postoperative pulmonary complications, with scores greater or equal than 45 indicating high risk.

* Defined as hemoglobin ≤ 10 g/dL

### Outcomes

Postoperative pulmonary complications occurred in 425 (35·9%) patients (Table 2). The most frequent postoperative pulmonary complication was mild respiratory failure (21·6%), followed by pleural effusion (14·3%) and pulmonary infection (10·7%). The incidence of PPCs of excluded patients because of missing data and of the two original trials are shown in eTable 3 and 4 in the Online Supplement. The distribution of postoperative pulmonary complications in the two original trials was different, as the incidence of pulmonary effusion and pulmonary infection was higher in the PROVHILO study compared to the PROBESE study (16·6% and 13·8%, versus 8·1% and 2·5%). However, the incidence of severe respiratory failure was higher in the PROBESE study compared to the PROVHILO study (11·5% versus 5·7%).

**Table 2:** Primary and secondary outcomes.

	Participants (n = 1191)
PPC – no (%)	425 (35·9)
Mild respiratory failure	255 (21·6)
Severe respiratory failure	86 (7·3)
Pleural effusion	169 (14·3)
Pulmonary infection	127 (10·7)
Atelectasis	101 (8·5)
Bronchospasm	43 (3·6)
Pneumothorax	15 (1·3)
ARDS	8 (0·7)
Aspiration	4 (0·3)

Data are median (quartile 25% – quartile 75%) or No (%). Percentages may not total 100 because of rounding.

PPC: postoperative pulmonary complication; ARDS: acute respiratory distress syndrome.

### Driving pressure and the mechanical power

**Figure 2:**
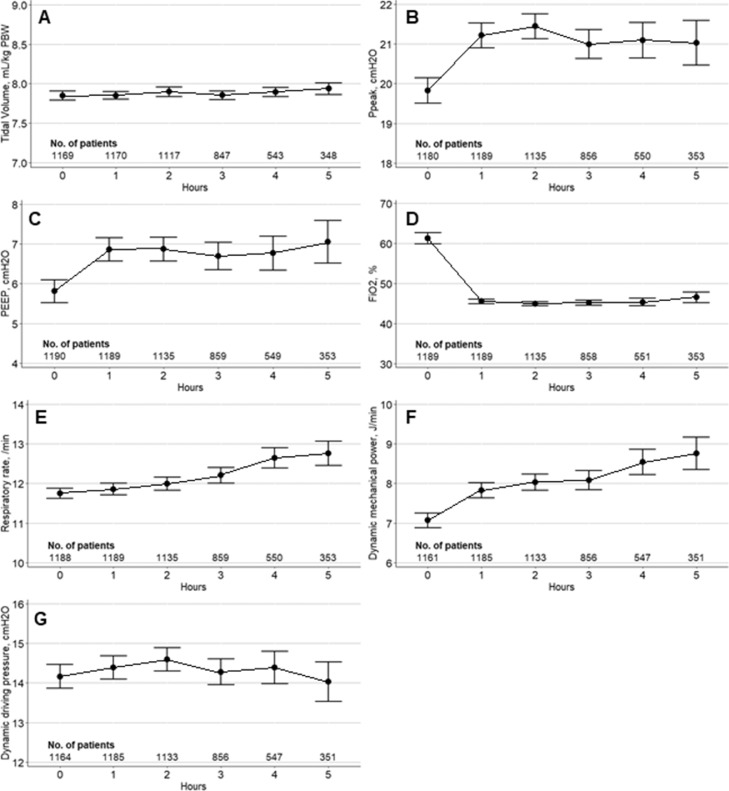
**Intraoperative ventilation parameters during the first five hours of surgery**. First row panels (A and B): mean hourly values of V_T_ and Ppeak. Second row panels (C and D): mean hourly values of PEEP and FiO_2_. Third row panels (E and F): mean hourly values of RR and MP. Fourth row panel (G): mean hourly ΔP values. Circles are means and error bars are 95% confidence intervals. The number of patients is presented below. PBW: predicted body weight; Ppeak: peak pressure; PEEP: positive end–expiratory pressure; FiO2: fraction of inspired oxygen

Ventilation parameters at the first hour of ventilation are presented in eTable 5 in the Online Supplement. Median tidal volume was 7·9 (7·3–8·1) mL/kg predicted body weight and median respiratory rate was 12 (10–13) /min at the first hour of ventilation. Median time–weighted average driving pressure and mechanical power were 14·0 [11·0–17·0] cmH_2_O, and 7·6 [5·1–10·0] J/min, respectively. Ventilator parameters, including driving pressure and the mechanical power, during the first 5 hours of ventilation are shown in respectively Figure 2, and for the two original studies in eFigure 2 in the Online Supplement. During surgery, an increase of intraoperative mechanical power, respiratory rate, PEEP and peak pressures in time is seen. No such trend of driving pressure is observed. Besides, intraoperative mechanical power is higher in patients from the PROBESE study compared to patients from the PROVHILO study. Median time–weighted driving pressure and the mechanical power in patients with and without occurrence of postoperative pulmonary complications were 13·7 [11·0–17·0] and 13·8 [11·0–17·0] cmH_2_O (*p*=0·997), and 7·9 [5·4–10·4] and 7·5 [4·9–9·8] J/min (*p*=0·026), respectively.

### Associations of driving pressure and the mechanical power with the occurrence of postoperative pulmonary complications

**Figure 3:**
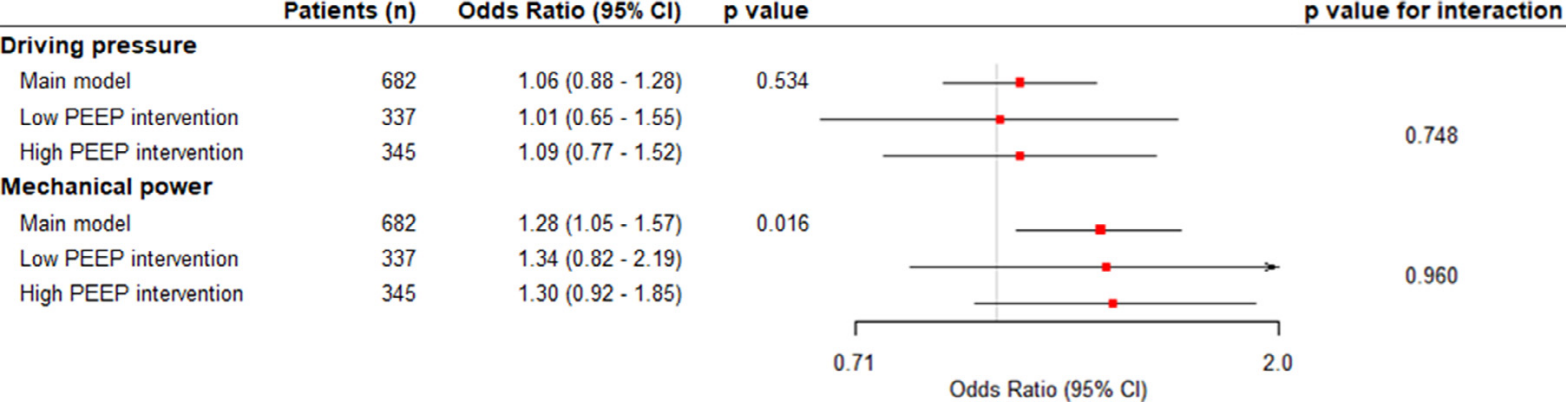
**Multivariable model assessing the association of time–weighted average driving pressure and mechanical power with postoperative pulmonary complications**. Models adjusted for age, gender, ASA classification, ARISCAT score, centre, history of COPD, history of active cancer, history of heart failure, SpO_2_, respiratory rate, heart rate, mean arterial blood pressure, fraction of inspired oxygen and end–tidal carbon dioxide in the first hour of intraoperative ventilation, antibiotic prophylaxis, duration of surgery, duration of anaesthesia, use of epidural, type of anaesthesia, amount of blood loss, total amount of fluid administered, transfusion, urine output, emergency procedure and trial. Odds ratios were the adjusted odds ratios associated with a 1–point increment. Values higher than 1 indicate an association with increased risk of postoperative pulmonary complications. CI: confidence interval; ΔP: driving pressure; MP: mechanical power; PEEP: positive end–expiratory pressure.

As no multicollinearity was found using gender, BMI, ASA classification score and ARISCAT score as covariates, driving pressure and mechanical power were used in the same model (Table 3). In the multivariate model, an increase of 1 cm H_2_O in driving pressure was not (odds ratio, 1·06 [95% CI 0·88–1·28], *p*=0.534), but an increase of 1 J/min in mechanical power was associated with occurrence of postoperative pulmonary complications after adjustment for all confounders (odds ratio, 1·28 [95% CI 1·05–1·57], *p*=0·016) (Figure 3). The complete regression model in shown in eTable 6 in the Online Supplement. The E–value for the odds ratio of mechanical power was 1.9 and for the confidence interval 1.28.

**Table 3:** Multivariable Model Assessing the Association of Driving Pressure and Mechanical Power with PPC in the Same Model.

	Odds Ratio(95% CI)	*p* value	VIF
Age	1·19 (1·02 – 1·39)	0·022	1·329
Male gender	0·98 (0·75 – 1·28)	0·886	1·080
Body mass index	0·99 (0·79 – 1·23)	0·908	1·522
ASA^†^ classification			1·224
1	Reference		
2	2·79 (1·61 – 5·12)	< 0·001	
3	4·04 (2·27 – 7·58)	< 0·001	
4	3·92 (1·17 – 13·46)	0·027	
ARISCAT score^‡^	1·29 (1·13 – 1·48)	< 0·001	1·189
Driving pressure	0·99 (0·86 – 1·13)	0·910	1·119
Mechanical power	1·28 (1·11 – 1·47)	< 0·001	1·303

VIF: variance inflation factor.

Odds ratio represents an one point increase of the variable.

† The American Society of Anesthesiologists (ASA) criteria for physical status include a classification for normal health (1), mild systemic disease (2), severe systemic disease (3), severe systemic disease that is a constant threat to life (4), and a moribund person who is not expected to survive without the operation (5).

‡ The Assess Respiratory Risk in Surgical Patients in Catalonia (ARISCAT) score estimates the risk of postoperative pulmonary complications, with scores greater or equal than 45 indicating high risk.

Variance inflation factor > 2.50 indicates potential multicollinearity.

### Sensitivity analyses

Repeating the primary analysis without imputation of missing data, did not change the main findings (eTable 7 in the Online Supplement). No interaction was found between driving pressure or mechanical power and PEEP intervention group, respectively *p*=0·748 and *p*=0·960 (Figure 3), meaning that the associations between the parameters for intensity of ventilation were not affected by treatment allocation, i.e., affected by the level of PEEP used. Besides, no interaction was found between driving pressure or mechanical power and body mass index, respectively *p*=0·803 and *p*=0·626.

## Discussion

In this posthoc analysis of two randomised controlled trials comparing intraoperative ventilation at two different levels of PEEP in patients at increased risk of postoperative pulmonary complications planned for major abdominal surgery, we found that higher mechanical power was, but higher driving pressure was not, independently associated with increased occurrence of postoperative pulmonary complications after adjustment for confounders. The association of the mechanical power with postoperative pulmonary complications was not affected by body mass index or treatment allocation, i.e., the level of PEEP used did not affect this finding.

Compared to other cohorts of abdominal surgery patients, our patient cohort had similar baseline characteristics.^4^^,^^7^ The incidence of postoperative pulmonary complications was 35·9%, which was high compared to other cohorts of patients undergoing abdominal surgery,^2^^,^^7^ but was similar to studies in patients with intermediate–high risk for postoperative pulmonary complications following the ARISCAT classification.^19^^,^^20^ Multiple studies on intraoperative driving pressure have reported similar driving pressure values,^5^^,^^8^^,^^24^ even in open abdominal surgery.^7^ Thus far, the intraoperative mechanical power has been reported in cardiothoracic, thoracic, and non–thoracic surgery patients and was slightly higher to that in our patient cohort, with median mechanical power of approximately 8–9 J/min.^24^, ^25^, ^26^ Comparing the two original studies, dynamic mechanical power in the PROBESE study was higher compared to the PROVHILO study, which could be attributed to a higher body mass index in these patients leading to lower chest wall compliance. Our study is the first to report on the trend of the intraoperative mechanical power showing an increase intraoperative mechanical power in time, without an increase of dynamic driving pressure. Parallel, the increase of respiratory rate and peak pressure in time could contribute to the increase of mechanical power. These changes could have various aetiologies, which could be studies in future research.

This study is the first to investigate both intraoperative driving pressure and the mechanical power in patients undergoing open abdominal surgery. In contrast to previous studies in patients undergoing intraoperative ventilation for abdominal and non–abdominal surgery, we found no significant association of intraoperative driving pressure with postoperative pulmonary complications.^5^, ^6^, ^7^, ^8^ Previous studies enrolled patients between 2004 and 2017,^5^^,^^6^^,^^8^ reporting driving pressure values with a wider distribution compared to the two merged trials for this analysis, that enrolled patients between 2011 and 2018. As driving pressure has been incorporated as a target in intraoperative ventilation management only in recent years, our study could have had an insufficient sample size to confirm the presence of an association of driving pressure with postoperative pulmonary complications. However, no such difference was found in comparison to a recent report on the association between driving pressure and postoperative pulmonary complications in patients undergoing open and laparoscopic abdominal surgery.^7^ In our current analysis, the intraoperative mechanical power after adjustment of confounders was independently associated with the occurrence of postoperative pulmonary complications, being in contrast to a study in cardiothoracic patients, in which both intraoperative driving pressure and mechanical power were not associated with postoperative pulmonary complications.^24^ One study assessed the mechanical power in one lung ventilation in thoracic surgery patients and reported that time–weighted mechanical power was higher in patients who later developed postoperative pulmonary complications.^25^ A recent study in patients undergoing major non–cardiothoracic, non–intracranial surgery showed that the mechanical power normalized for respiratory system compliance was independently associated with the incidence of postoperative pulmonary complications.^26^ Of note, only one measurement, the highest one, of the intraoperative mechanical power was considered. In our sensitivity analyses, we showed that the found associations were independent of body mass index and the PEEP level used. Surprisingly, no association between the mechanical power and postoperative pulmonary complications was found in the high PEEP group, given the contribution of PEEP to the mechanical power.

Despite the absence of an association between intraoperative driving pressure and postoperative pulmonary complications, driving pressure remains a valuable digital biomarker. Driving pressure has been found to be associated with patient outcome in both intraoperative ventilation^5^, ^6^, ^7^, ^8^ and ventilation in critically ill patients.^15^^,^^21^ Recently, a single–centre randomised clinical trial showed that using driving pressure–guided individualized PEEP levels resulted in less clinically significant postoperative pulmonary complications compared to using a fixed PEEP in patients undergoing open upper abdominal surgery.^27^ These findings should be verified in future randomised clinical trials. The absence of an association between driving pressure and the occurrence of postoperative pulmonary complications in our study could be due to a lack of statistical power. Our results indicate that the mechanical power could serve as an additional digital biomarker to driving pressure that could guide clinicians to monitor the dynamic strain of the aerated lung tissue. To aid clinicians, driving pressure and the mechanical power and trends thereof could be visualized on ventilator screens, possibly even with safety boundaries. It could be very complex to reduce or minimize the mechanical power, as it depends on changes in 4 different ventilator settings that could even have opposite effect–for instance, a reduction in tidal volume size reduces the mechanical power, but the higher respiratory rate to compensate for the lower minute ventilation actually increases it. Automated systems could be helpful, as recently suggested in a study in critically ill patients with acute hypoxemic respiratory failure.^28^ Furthermore, limiting the intensity of ventilation by reducing intraoperative driving pressure and mechanical power is a promising strategy to minimize ventilator–induced lung injury and improve patient outcome. As it is still unknown if this association just follows the relationship between respiratory system compliance and patient outcome, randomised clinical trials remain needed to verify the effect of minimizing the intensity of ventilation on patient outcomes.

Many equations for calculating the mechanical power in an individual patient have been suggested and used.^9^^,^^15^^,^^22^^,^^29^^,^^30^ As transpulmonary and plateau pressures are not routinely measured, we used dynamic driving pressure in the mechanical power equation. This substitution is both practical and reliable,^22^ and others validated this approach.^15^^,^^16^

This analysis has strengths. This is the first study to report on the association between the exposure to the mechanical power and postoperative pulmonary complications in patients undergoing open abdominal surgery using longitudinal data collected at fixed timepoints. Patients undergoing laparoscopic abdominal surgery were excluded, as pneumoperitoneum influences intraoperative ventilation due to higher intra–abdominal pressure. The combined international studies were conveniently sized, recently performed, conducted in a short period of time and included a large number of academic and non–academic centres worldwide, increasing robustness and generalizability of the results. We also followed a preplanned analysis plan. Besides, the characteristics of the two original trials were provided.

This study also has limitations. The included data was the result of merging two randomised clinical trials, one could question the generalizability for regular intraoperative care. However, the sensitivity analysis showed no differences in parameters of intensity of ventilation between PEEP intervention groups. Besides, all patients had an increased risk for postoperative pulmonary complications and underwent open abdominal surgery and were mechanically ventilated following endotracheal intubation. This could decrease generalizability in patients with a lower risk for postoperative pulmonary complications, undergoing laparoscopic abdominal or non–abdominal surgery, or when using supraglottic airway devices. Also, in post–hoc analyses a form of residual confounding cannot be excluded. Unfortunately, important factors that could also have an association with the outcome of interest, like fluid balance and history of valve disease, were not collected in the original studies. Tube size was also not recorded in the two studies. Furthermore, the use of a composite endpoint could be seen as a limitation. However, the incidence of the separate postoperative pulmonary complications was reported and even minor pulmonary complications, like unplanned supplementary oxygen, are associated with clinically relevant outcomes, like duration of hospital stay.^2^ Moreover, the correlation between transpulmonary pressure and peak pressure in calculating driving pressure could be distorted during open abdominal surgery. As driving pressure is used in the mechanical power equation, both driving pressure and the mechanical power could be influenced by this possible distortion. Lastly, despite a recent report that mechanical power normalized for predicted body weight or respiratory system compliance was prognostic superior to absolute mechanical power in the critically–ill,^11^ the absolute mechanical power was assessed in this study being more validated compared to the normalized mechanical power.

In this merged cohort of surgery patients at an increased risk for postoperative pulmonary complications that were planned for open abdominal surgery, higher mechanical power was independently associated with postoperative pulmonary complications. The mechanical power could serve as a summary ventilatory in these patients, but our findings needs confirmation in other, preferably prospective studies.

## Declaration of interests

Marcelo Gama de Abreu declares consultations at Ambu, ZOLL, Lungpacer and patents on variable pressure support. Serpa Neto reports lecture fees from Dräger, outside of the submitted work. Schultz reports personal fees from Hamilton (In 2018, Marcus Schultz attended a workshop organized by Hamilton, in which expenses for lodging were covered for all invited experts, participants from abroad had their travel expenses reimbursed, and speakers received a speaker's fee of CHF 800 – this has no relation with the current study. Additionally, Marcus Schultz’ team uses so-called ‘memory boxes’ for capturing ventilation data – these boxes are lent for free, and will be send back at the end of the study for which they are used – this has no relation with the current study) outside of the submitted work. All other authors declare no competing interests.

## Appendix Supplementary materials

Image, application 1

Image, application 2

## Footnotes

Supplementary material associated with this article can be found in the online version at doi:10.1016/j.eclinm.2022.101397.
