# Supplementary material for: Associations of dynamic driving pressure and mechanical power with postoperative pulmonary complications–posthoc analysis of two randomised clinical trials in open abdominal surgery
Source: eClinicalMedicine. 2022 Apr 16;47:101397. doi: 10.1016/j.eclinm.2022.101397 (PMC9035701; doi:10.1016/j.eclinm.2022.101397)
Supplement: Supplementary file 1 [file mmc1.docx]

**ONLINE SUPPLEMENT with:**

**Associations of Dynamic Driving Pressure and Mechanical Power with Postoperative Pulmonary Complications––posthoc analysis of two randomised clinical trials in open abdominal surgery**

**TABLE OF CONTENT**

**Definitions of pulmonary post–operative complications 3**

**eTable 14**

**eTable 26**

**eTable 38**

**eTable 49**

**eTable 510**

**eTable 611**

**eTable 712**

**eFigure 113**

**eFigure 214**

**Collaborators of the two original studies15**

**Definitions of pulmonary post–operative complications**

Bronchospasm:

Defined as newly detected expiratory wheezing treated with bronchodilators

Respiratory failure:

Mild: PaO_2_ < 60 mmHg or SpO_2_ < 90% in room air during at least 10 min in need of supplemental oxygen (excluding hypoventilation) *and* Severe: PaO_2_ < 60 mmHg or SpO_2_ < 90% despite supplemental oxygen or in need for non–invasive or invasive mechanical ventilation (excluding hypoventilation)

Acute respiratory distress syndrome:

Mild, moderate or severe according to the Berlin Definition for ARDS

Pulmonary infection:

Defined as new or progressive radiographic infiltrate plus at least two of the following: antibiotic treatment, tympanic temperature > 38ªC, leukocytosis or leucopenia (WBC count < 4,000cells/mm3 or > 12,000cells/mm3) and/or purulent secretions

Atelectasis:

Suggested by lung opacification with shift of the mediastinum, hilum, or hemidiaphragm towards the affected area, and compensatory overinflation in the adjacent non–atelectatic lung

Pleural effusion:

Chest X–ray demonstrating blunting of the costophrenic angle, loss of the sharp silhouette of the ipsilateral hemidiaphragm in upright position, evidence of displacement of adjacent anatomical structures, or (in supine position) a hazy opacity in one hemithorax with preserved vascular shadows

Pneumothorax:

Defined as air in the pleural space with no vascular bed surrounding the visceral pleura

| **eTable 1 – Baseline patient characteristics of eligible patients from the original trials, included patients and excluded patients due to missing data** | | | | |
| --- | --- | --- | --- | --- |
|  | **Eligible patients from the original trials (n = 1627)** | **Included patients**  **(n = 1191)** | **Excluded patients due to missing data (n = 436)** | ***p* value** |
| Age – years | 64 (54 – 73) | 65 (57 – 72) | 68·0 (58·0 – 75·0) | <0·001 |
| Male gender – no (%) | 707 (43·6) | 568 (47·7) | 139 (32·3) | <0·001 |
| Body mass index – kg/m^2^ | 26·7 (23·6 – 32·0) | 27·1 (23·9 – 35·3) | 25·8 (23·0 – 28·7) | <0·001 |
| ASA^†^ classification – no (%) |  |  |  | 0·003 |
| 1 | 132 (8·2) | 114 (9·6) | 18 (4·2) |  |
| 2 | 774 (47·9) | 572 (48·1) | 202 (47·5) |  |
| 3 | 686 (42·5) | 489 (41·1) | 197 (46·4) |  |
| 4 | 22 (1·4) | 15 (1·3) | 7 (1·6) |  |
| 5 | 1 (0·1) | 0 (0·0) | 1 (0·2) |  |
| ARISCAT score^‡^ | 41 (34 – 42) | 41 (34 – 44) | 38 (31 – 41) | <0·001 |
| ARISCAT^‡^ classification |  |  |  | <0·001 |
| Intermediate – no (%) | 1275 (78·4) | 906 (76·1) | 369 (84·6) |  |
| High – no (%) | 352 (21·6) | 285 (23·9) | 67 (15·4) |  |
| Risk factors for PPC – no (%) |  |  |  |  |
| SpO_2_ | 97 (96 – 98) | 97 (96 – 98) | 98 (96 – 99) | <0·001 |
| SpO_2_ classification |  |  |  | 0·616 |
| ≥ 96 | 1259 (78·5) | 924 (77·9) | 335 (80·1) |  |
| 91 – 95 | 330 (20·6) | 251 (21·2) | 79 (18·9) |  |
| < 91 | 15 (0·9) | 11 (0·9) | 4 (1·0) |  |
| Respiratory infection within the last month | 82 (5·1) | 66 (5·5) | 16 (3·7) | 0·140 |
| Anemia* | 165 (10·6) | 108 (9·5) | 57 (13·5) | 0·031 |
| Emergency procedure | 41 (2·5) | 40 (3·4) | 1 (0·2) | <0·001 |
| Coexisiting disorders – no (%) |  |  |  |  |
| Heart failure | 220 (13·7) | 196 (16·7) | 24 (5·5) | <0·001 |
| COPD | 115 (7·1) | 92 (7·7) | 23 (5·3) | 0·088 |
| Active cancer | 1002 (61·8) | 642 (54·1) | 360 (82·6) | <0·001 |
| Surgical approach – no (%) |  |  |  | **–** |
| Non–laparoscopic | 1627 (100) | 1191 (100) | 436 (100) |  |
| Laparoscopic | 0 (0) | 0 (0) | 0 (0·0) |  |
| Type of surgery – no (%) |  |  |  | – |
| Abdominal | 1627 (100) | 1191 (100) | 436 (100) |  |
| Non abdominal | 0 (0) | 0 (0·0) | 0 (0·0) |  |
| Duration of surgery – minutes | 200 (145 – 280) | 190 (138 – 276) | 220 (170 – 280) | <0·001 |
| Specific procedure – no (%) |  |  |  | <0·001 |
| Bariatric | 25 (1·5) | 24 (2·0) | 1 (0·2) |  |
| Bladder or urologic | 152 (9·3) | 133 (11·2) | 19 (4·4) |  |
| Bowel | 41 (2·5) | 32 (2·7) | 9 (2·1) |  |
| Colorectal | 443 (27·2) | 322 (27·0) | 121 (27·8) |  |
| Gastric | 144 (8·9) | 94 (7·9) | 50 (11·5) |  |
| Gynecology | 102 (6·3) | 94 (7·9) | 8 (1·8) |  |
| Hepatic | 208 (12·8) | 119 (10·0) | 89 (20·4) |  |
| Hernia | 50 (3·1) | 42 (3·5) | 8 (1·8) |  |
| Kidney | 30 (1·8) | 25 (2·1) | 5 (1·1) |  |
| Pancreatic | 202 (12·4) | 128 (10·7) | 74 (17·0) |  |
| Vascular | 50 (3·1) | 37 (3·1) | 13 (3·0) |  |
| Other | 180 (11·1) | 141 (11·8) | 39 (8·9) |  |
| Data are median (quartile 25% – quartile 75%) or No (%). Percentages may not total 100 because of rounding.  *P* values are calculated comparing included patients and patients excluded due to missing data.  *PEEP: positive end expiratory pressure; ARISCAT: Assess Respiratory Risk in Surgical Patients in Catalonia; ASA: American Society of Anesthesiology; PPC: postoperative pulmonary complications; COPD: chronic obstructive pulmonary disease.*  † The American Society of Anesthesiologists (ASA) criteria for physical status include a classification for normal health (1) – mild systemic disease (2) – severe systemic disease (3) – severe systemic disease that is a constant threat to life (4) – and a moribund person who is not expected to survive without the operation (6).  ‡ The Assess Respiratory Risk in Surgical Patients in Catalonia (ARISCAT) score estimates the risk of postoperative pulmonary complications – with scores greater or equal than 45 indicating high risk.  * Defined as hemoglobin ≤ 10 g/dL | | | | |

| **eTable 2 – Baseline patient characteristics per (original) trial** | | | |
| --- | --- | --- | --- |
|  | **All Participants**  **(n = 1191)** | **PROVHILO**  **(n = 869)** | **PROBESE**  **(n = 322)** |
| Age, years | 65 (57 – 72) | 66 (55 – 72) | 56 (48 – 65) |
| Male gender – no (%) | 568 (47·7) | 370 (42·6) | 198 (61·5) |
| Body mass index, kg/m^2^ | 27·1 (23·9 – 35·3) | 25·5 (22·7 – 28·0) | 39·2 (36·6 – 43·7) |
| ASA^†^ classification – no (%) |  |  |  |
| 1 | 114 (9·6) | 106 (12·2) | 8 (2·5) |
| 2 | 572 (48·1) | 465 (53·6) | 107 (33·2) |
| 3 | 489 (41·1) | 286 (32·9) | 203 (63·0) |
| 4 | 15 (1·3) | 11 (1·3) | 4 (1·2) |
| ARISCAT score^‡^ | 41 (34 – 44) | 41 (34 – 44) | 39 (34 – 42) |
| Intermediate – no (%) | 906 (76·1) | 658 (75·7) | 248 (77·0) |
| High – no (%) | 285 (23·9) | 211 (24·3) | 74 (23·0) |
| Risk factors for PPC – no (%) |  |  |  |
| SpO_2_ | 97 (96 – 98) | 97 (96 – 98) | 96 (95 – 97) |
| ≥ 96 | 924 (77·9) | 720 (83·3) | 204 (63·4) |
| 91 – 95 | 251 (21·2) | 138 (16·0) | 113 (35·1) |
| < 91 | 11 (0·9) | 6 (0·7) | 5 (1·6) |
| Respiratory infection within the  last month | 66 (5·5) | 48 (5·5) | 18 (5·6) |
| Anemia* | 108 (9·5) | 82 (9·7) | 26 (9·0) |
| Emergency procedure | 40 (3·4) | 16 (1·8) | 24 (7·5) |
| Coexisiting disorders – no (%) |  |  |  |
| Heart failure | 196 (16·7) | 188 (22·1) | 8 (2·5) |
| COPD | 92 (7·7) | 67 (7·7) | 25 (7·8) |
| Active cancer | 642 (54·1) | 530 (61·3) | 112 (34·8) |
| Surgical approach – no (%) |  |  |  |
| Non–laparoscopic | 1191 (100) | 869 (100) | 322 (100) |
| Laparoscopic | 0 (0) | 0 (0) | 0 (0) |
| Type of surgery – no (%) |  |  |  |
| Abdominal | 1191 (100) | 869 (100) | 322 (100) |
| Non abdominal | 0 (0) | 0 (0) | 0 (0) |
| Duration of surgery – minutes | 190 (138 – 276) | 200 (140 – 290) | 180 (132 – 240) |
| Specific procedure – no (%) |  |  |  |
| Bariatric | 24 (2·0) | 0 (0·0) | 24 (7·5) |
| Bladder or urologic | 133 (11·2) | 109 (12·5) | 24 (7·5) |
| Bowel | 32 (2·7) | 22 (2·5) | 10 (3·1) |
| Colorectal | 322 (27·0) | 285 (32·8) | 37 (11·5) |
| Gastric | 94 (7·9) | 84 (9·7) | 10 (3·1) |
| Gynecology | 94 (7·9) | 42 (4·8) | 52 (16·1) |
| Hepatic | 119 (10·0) | 89 (10·2) | 30 (9·3) |
| Hernia | 42 (3·5) | 6 (0·7) | 36 (11·2) |
| Kidney | 25 (2·1) | 22 (2·5) | 3 (0·9) |
| Pancreatic | 128 (10·7) | 119 (13·7) | 9 (2·8) |
| Vascular | 37 (3·1) | 34 (3·9) | 3 (0·9) |
| Other | 141 (11·8) | 57 (6·6) | 84 (26·1) |
| Data are median (quartile 25% – quartile 75%) or No (%). Percentages may not total 100 because of rounding  *PEEP: positive end expiratory pressure; ARISCAT: Assess Respiratory Risk in Surgical Patients in Catalonia; ASA: American Society of Anesthesiology; PPC: postoperative pulmonary complications.*  † The American Society of Anesthesiologists (ASA) criteria for physical status include a classification for normal health (1), mild systemic disease (2), severe systemic disease (3), severe systemic disease that is a constant threat to life (4), and a moribund person who is not expected to survive without the operation (6).  ‡ The Assess Respiratory Risk in Surgical Patients in Catalonia (ARISCAT) score estimates the risk of postoperative pulmonary complications, with scores greater or equal than 45 indicating high risk.  * Defined as hemoglobin ≤ 10 g/dL | | | |

| **eTable 3 – Primary and secondary outcomes of eligible patients from the original trials, included patients and excluded patients due to missing data** | | | | |
| --- | --- | --- | --- | --- |
|  | **Eligible patients from the original trials (n = 1627)** | **Included patients**  **(n = 1191)** | **Excluded patients due to missing data (n = 436)** | ***p* value** |
| PPC – no (%) | 642 (39·8) | 425 (35·9) | 217 (50·5) | <0·001 |
| Mild respiratory failure | 376 (23·) | 255 (21·6) | 121 (28·1) | 0·006 |
| Severe respiratory failure | 122 (7·6) | 86 (7·3) | 36 (8·4) | 0·742 |
| Pleural effusion | 251 (15·6) | 169 (14·3) | 82 (19·0) | 0·020 |
| Pulmonary infection | 144 (8·9) | 127 (10·7) | 17 (3·9) | <0·001 |
| Atelectasis | 227 (14·1) | 101 (8·5) | 126 (29·2) | <0·001 |
| Bronchospasm | 46 (2·9) | 43 (3·6) | 3 (0·7) | 0·002 |
| Pneumothorax | 18 (1·1) | 15 (1·3) | 3 (0·7) | 0·333 |
| ARDS | 24 (1·5) | 8 (0·7) | 16 (3·7) | <0·001 |
| Aspiration | 4 (0·3) | 4 (0·3) | 0 (0·0) | 0·805 |
| Data are median (quartile 25% – quartile 75%) or No (%). Percentages may not total 100 because of rounding  *P* values are calculated comparing included patients and patients excluded due to missing data.  *PPC: postoperative pulmonary complication; ARDS: acute respiratory distress syndrome.* | | | | |

| **eTable 4 – Primary and secondary outcomes per (original) trial** | | | |
| --- | --- | --- | --- |
|  | **All Participants**  **(n = 1191)** | **PROVHILO**  **(n = 869)** | **PROBESE**  **(n = 322)** |
| PPC – no (%) | 425 (35·9) | 315 (36·6) | 110 (34·2) |
| Mild respiratory failure | 255 (21·6) | 187 (21·8) | 68 (21·1) |
| Severe respiratory failure | 86 (7·3) | 49 (5·7) | 37 (11·5) |
| Pleural effusion | 169 (14·3) | 143 (16·6) | 26 (8·1) |
| Pulmonary infection | 127 (10·7) | 119 (13·8) | 8 (2·5) |
| Atelectasis | 101 (8·5) | 76 (8·8) | 25 (7·8) |
| Bronchospasm | 43 (3·6) | 35 (4·1) | 8 (2·5) |
| Pneumothorax | 15 (1·3) | 13 (1·5) | 2 (0·6) |
| ARDS | 8 (0·7) | 7 (0·8) | 1 (0·3) |
| Aspiration | 4 (0·3) | 4 (0·5) | 0 (0) |
| Data are median (quartile 25% – quartile 75%) or No (%). Percentages may not total 100 because of rounding  *PPC: postoperative pulmonary complication; ARDS: acute respiratory distress syndrome.* | | | |

| **eTable 5 – Ventilator parameters at the first hour of ventilation per (original) trial** | | | |
| --- | --- | --- | --- |
|  | **All Participants**  **(n = 1191)** | **PROVHILO**  **(n = 869)** | **PROBESE**  **(n = 322)** |
| Tidal volume, mL | 480·0 (420·0 – 540·0) | 500·0 (450·0 – 550·0) | 418 (375·0 – 478·8) |
| Tidal volume, mL/kg PBW | 7·9 (7·3 – 8·1) | 8·0 (7·9 – 8·2) | 7·0 (7·0 – 7·2) |
| PEEP, cmH_2_O | 5·0 (2·0 – 12·0) | 5·0 (2·0 – 12·0) | 8·5 (4·0 – 12·0) |
| Respiratory rate, mpm | 12 (10 – 13) | 11 (10 – 12) | 14 (12 – 16) |
| Peak pressure, cmH_2_O | 22·0 (17·0 – 25·0) | 20·0 (16·0 – 24·0) | 24·0 (22·0 – 27·0) |
| Driving pressure, cmH_2_O | 14·0 (11·0 – 17·0) | 13·0 (10·0 – 16·0) | 16·0 (12·0 – 20·0) |
| Mechanical power, J/min | 7·6 (5·1 – 10·0) | 6·7 (4·5 – 9·3) | 9·2 (7·6 – 11·4) |
| FiO_2_, % | 41 (40 – 50) | 40 (40 – 50) | 50 (40 – 50) |
| EtCO_2_, mmHg | 35·0 (33·0 – 37·6) | 35·0 (32·0 – 37·0) | 37·0 (35·0 – 40·0) |
| *PPW: predicted body weight; PEEP:* *positive end–expiratory pressure; FiO_2:_* *fraction of inspired oxygen_;_ EtCO_2:_ End*–*tidal carbon dioxide* | | | |

| **eTable 6 – Factors associated with the occurrence of postoperative pulmonary complications** | | |
| --- | --- | --- |
|  | **Multivariable Analysis** | |
|  | **Odds Ratio**  **(95% CI)** | ***p* value** |
| **Demographic characteristics** |  |  |
| Age (years) | 1·22 (0·98 – 1·53) | 0·083 |
| Male gender | 1·18 (0·81 – 1·73) | 0·390 |
| Body mass index (kg/m²) | 0·90 (0·65 – 1·23) | 0·512 |
| ASA classification |  |  |
| 1 |  |  |
| 2 | 2·90 (1·30 – 6·92) | 0·012 |
| 3 | 4·40 (1·90 – 10·92) | 0·001 |
| 4 | 7·21 (1·20 – 60·98) | 0·041 |
| ARISCAT score | 1·06 (0·86 – 1·30) | 0·592 |
| **Co–existing disorders** |  |  |
| Active cancer | 0·74 (0·50 – 1·10) | 0·139 |
| Heart failure | 1·41 (0·82 – 2·42) | 0·215 |
| COPD | 1·83 (0·99 – 3·42) | 0·056 |
| **Pre–operative vital signs** |  |  |
| Heart rate (beats/min) | 1·13 (0·65 – 1·35) | 0·171 |
| Mean arterial pressure (mmHg) | 0·96 (0·81 – 1·14) | 0·670 |
| Respiratory rate (breaths/min) | 0·93 (0·78 – 1·12) | 0·457 |
| SpO_2_ (%) | 0·80 (0·66 – 0·99) | 0·037 |
| **Intraoperative ventilation parameters** |  |  |
| FiO_2_ (%) | 1·02 (0·86 – 1·21) | 0·826 |
| EtCO_2_ | 1·02 (0·84 – 1·23) | 0·852 |
| Time–weighted driving pressure (cmH_2_O) | 1·06 (0·88 – 1·28) | 0·534 |
| Time–weighted mechanical power (J/min) | 1·28 (1·05 – 1·57) | 0·016 |
| **Other intraoperative parameters** |  |  |
| Antibiotic prophylaxis | 0·80 (0·36 – 1·81) | 0·585 |
| Emergency procedure | 1·20 (0·47 – 2·98) | 0·702 |
| Duration of surgery (min) | 1·01 (0·54 – 1·92) | 0·966 |
| Duration of anaesthesia (min) | 1·54 (0·80 – 3·02) | 0·202 |
| Use of epidural | 1·51 (1·03 – 2·23) | 0·034 |
| Type of anaesthesia | 1·26 (0·71 – 2·28) | 0·433 |
| Amount of blood loss (ml) | 1·29 (0·93 – 1·87) | 0·158 |
| Total amount of fluid administered (ml) | 1·29 (1·00 – 1·67) | 0·058 |
| Urinary output (ml) | 0·90 (0·75 – 1·10) | 0·287 |
| Any transfusion given | 1·16 (0·69 – 1·96) | 0·572 |
| Odds ratios of continuous covariates are given for a 1 unit increase of the covariate. Odds ratios higher than 1 indicate a positive association between the covariate and the occurrence of postoperative pulmonary complication. The model is adjusted for site and trial  *CI: confidence interval; ARISCAT: Assess Respiratory Risk in Surgical Patients in Catalonia; ASA: American Society of Anesthesiology; COPD: chronic obstructive pulmonary disease. SpO_2_: peripheral capillary oxygen saturation; FiO_2_: fraction of inspired oxygen; EtCO2: End–tidal carbon dioxide.* | | |

| **eTable 7 – Factors associated with the occurrence of postoperative pulmonary complications without imputation of missing data** | | |
| --- | --- | --- |
|  | **Multivariable Analysis** | |
|  | **Odds Ratio**  **(95% CI)** | ***p* value** |
| **Demographic characteristics** |  |  |
| Age (years) | 1·12 (0·89 – 1·43) | 0·332 |
| Male gender | 1·29 (0·87 – 1·92) | 0·209 |
| Body mass index (kg/m²) | 0·85 (0·61 – 1·18) | 0·342 |
| ASA classification |  |  |
| 1 |  |  |
| 2 | 3·34 (1·36 – 9·27) | 0·013 |
| 3 | 5·12 (2·12 – 14·59) | 0·001 |
| 4 | 6·87 (1·02 – 63·04) | 0·058 |
| ARISCAT score | 1·07 (0·86 – 1·33) | 0·555 |
| **Co–existing disorders** |  |  |
| Active cancer | 0·83 (0·55 – 1·25) | 0·378 |
| Heart failure | 1·54 (0·87 – 2·72) | 0·136 |
| COPD | 1·91 (1·02 – 3·60) | 0·043 |
| **Pre–operative vital signs** |  |  |
| Heart rate (beats/min) | 1·14 (0·95 – 1·38) | 0·154 |
| Mean arterial pressure (mmHg) | 0·94 (0·79 – 1·13) | 0·528 |
| Respiratory rate (breaths/min) | 0·92 (0·76 – 1·11) | 0·372 |
| SpO_2_ (%) | 0·79 (0·64 – 0·97) | 0·029 |
| **Intraoperative ventilation parameters** |  |  |
| FiO_2_ (%) | 1·05 (0·88 – 1·26) | 0·568 |
| EtCO_2_ | 1·03 (0·85 – 1·25) | 0·764 |
| Time–weighted driving pressure (cmH_2_O) | 1·01 (0·83 – 1·23) | 0·889 |
| Time–weighted mechanical power (J/min) | 1·31 (1·06 – 1·63) | 0·015 |
| **Other intraoperative parameters** |  |  |
| Antibiotic prophylaxis | 0·73 (0·33 – 1·66) | 0·442 |
| Emergency procedure | 1·28 (0·49 – 3·25) | 0·609 |
| Duration of surgery (min) | 0·72 (0·36 – 1·43) | 0·347 |
| Duration of anaesthesia (min) | 2·32 (1·12 – 4·86) | 0·024 |
| Use of epidural | 1·41 (0·94 – 2·11) | 0·096 |
| Type of anaesthesia | 1·28 (0·71 – 2·34) | 0·411 |
| Amount of blood loss (ml) | 1·25 (0·87 – 1·87) | 0·257 |
| Total amount of fluid administered (ml) | 1·25 (0·95 – 1·66) | 0·113 |
| Urinary output (ml) | 0·93 (0·76 – 1·15) | 0·478 |
| Any transfusion given | 1·39 (0·79 – 2·42) | 0·251 |
| Odds ratios of continuous covariates are given for a 1 unit increase of the covariate. Odds ratios higher than 1 indicate a positive association between the covariate and the occurrence of postoperative pulmonary complication. The model is adjusted for site and trial  *CI: confidence interval; ARISCAT: Assess Respiratory Risk in Surgical Patients in Catalonia; ASA: American Society of Anesthesiology; COPD: chronic obstructive pulmonary disease. SpO_2_: peripheral capillary oxygen saturation; FiO_2_: fraction of inspired oxygen; EtCO2: End–tidal carbon dioxide.* | | |

**eFigure 1 – Extended CONSORT diagram**

**
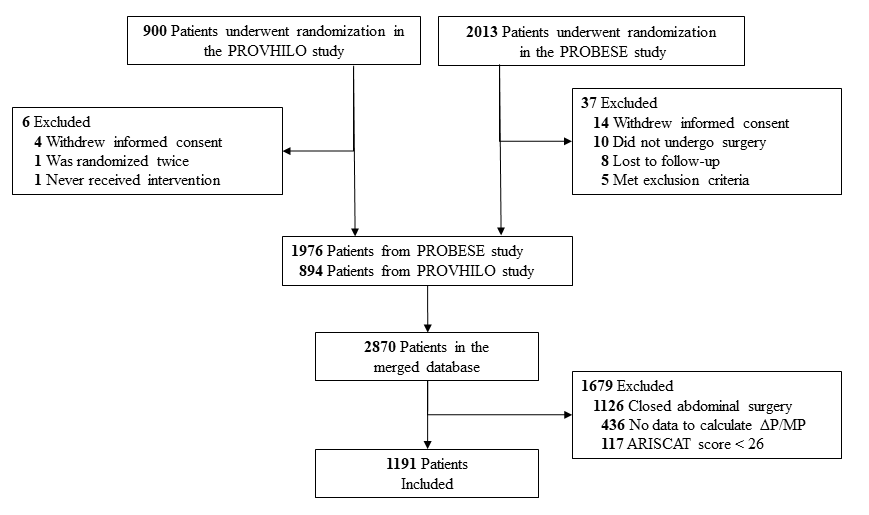
**

PROBESE: Protective Intraoperative Ventilation With Higher Versus Lower Levels of PEEP in Obese Patients; PROBESE: Protective Intraoperative Ventilation With Higher Versus Lower Levels of PEEP in Obese Patients; ΔP: driving pressure; MP: mechanical power. ARISCAT: Assess Respiratory Risk in Surgical Patients in Catalonia

**eFigure 2 – Intraoperative Ventilation Parameters During the First Five Hours of Surgery per original trial**

**
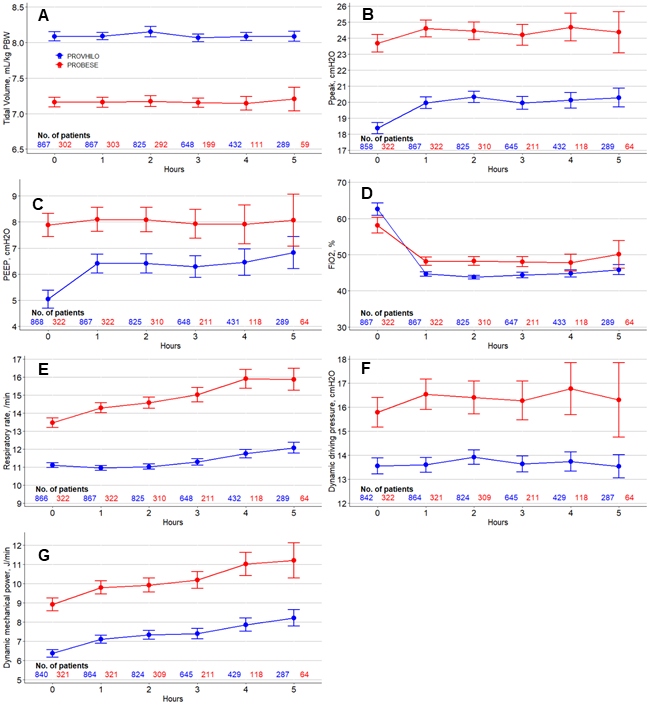
**

Data shown in red originates from the PROBESE trial and data shown in blue from the PROVHILO trial. First row panels (A and B): mean hourly values of tidal volume and peak pressure. Second row panels (C and D): mean hourly values of PEEP and FiO2. Third row panels (E and F): mean hourly values of RR and ΔP. Fourth row panel (G): mean hourly MP values. Circles are means and error bars are 95% confidence intervals. The number of patients is presented below. PBW: predicted body weigth; Ppeak: peak pressure; PEEP: positive end–expiratory pressure; FiO2: fraction of inspired oxygen

**PROVHILO Collaborative Group: Sabrine N T Hemmes, Marcelo Gama de Abreu, Paolo Severgnini, Markus W Hollmann, Jan M Binnekade, Hermann Wrigge, Jaume Canet, Michael Hiesmayr, Werner Schmid, Samir Jaber, Göran Hedenstierna, Christian Putensen, Paolo Pelosi, Marcus J Schultz, Jan M Binnekade, Daniel I Sessler, Burkhard Lachmann, Robert M Kacmarek, Arthur S Slutsky, Werner Schmid, Luc De Baerdemaeker, Stefan De Hert, Bjorn Heyse, Jurgen Van Limmen, Jan-Paul Mulier, David Velghe, Luc Jamaer, Jeroen Vandenbrande, Guillermo Bugedo, Jorge Florez, Tatjana Goranović, Branka Mazul-Sunko, Thomas Bluth, Marcelo Gama de Abreu, Andreas Güldner, Thomas Kiss, Thea Koch, Peter Markus Spieth, Christopher Uhlig, Jonathan Yaqub, Bea Bastin, Johann Geib, Maximilian S Schaefer, Martin Weiss, Tanja A Treschan, Andreas W Reske, Philipp Simon, Hermann Wrigge, Alexander Brodhun, Marion Ferner, Eric Hartmann, Rita Laufenberg-Feldmann, Lydia Strys, Christian Putensen, Edoardo De Robertis, Valter Perilli, Rodolfo Proietti, Bruno Amantea, Santo Caroleo, Francesco Tropea, Alessandro Bacuzzi, Paolo Severgnini, Massimo Vanoni, Gilda Cinnella, Girolamo Caggianelli, Davide D'Antini, Daniela La Bella, Giuseppina Mollica, Andrea Cortegiani, Antonino Giarratano, Francesca Montalto, Santi Maurizio Raineri, Bruno Barberis, Cristian Celentano, Michele Grio, Luigi Spagnolo, Angelo Gratarola, Alexandre Molin, Giulia Pellerano, Stefano Pezzato, Roberta Rusca, Giorgio Della Rocca, Lieuwe D J Bos, Sabrine N T Hemmes, Markus W Hollmann, Marcus J Schultz, Andrea Brunelli, Agnes Marti, Virginia Cegarra, Alfred Merten, Ma Victoria Moral, Ana Parera, Ma Carmen Unzueta, Sergi Sabaté, Pilar Sierra, Juan F Mayoral, Mercè Prieto, Manuel Granell Gil, Conrado Minguez Marín, Gary H Mills, Phoebe Bodger, Marcos F Vidal Melo, Demet Sulemanji, Juraj Sprung**

**PROBESE Collaborative Group:***Steering Committee:* Thomas Bluth, Ilona Bobek, Jaume C. Canet, Gilda Cinella, Luc de Baerdemaeker, Cesare Gregoretti, Göran Hedenstierna, Sabrine N. T. Hemmes, Michael Hiesmayr, Markus W. Hollmann, Samir Jaber, John Laffey, Marc-Joseph Licker, Klaus Markstaller, Idit Matot, Gary H. Mills, Jan Paul Mulier, Christian Putensen, Rolf Rossaint, Jochen Schmitt, Mert Senturk, Paolo Severgnini, Juraj Sprung, Marcos Francisco Vidal Melo, Hermann Wrigge, Ary Serpa Neto, Marcus J. Schultz, Paolo Pelosi, and Marcelo Gama de Abreu (chair). *Study Coordinating Center:* Thomas Bluth and Marcelo Gama de Abreu. *PROBESE Investigators:* The names are listed in alphabetical order. Fernando Abelha, Sühayla Abitağaoğlu, Marc Achilles, Afeez Adebesin, Ine Adriaensens, Charles Ahene, Fatima Akbar, Mohammed Al Harbi, Rita Al Khoury al Kallab, Xavier Albanel, Florence Aldenkortt, Rawan Abdullah Saleh Alfouzan, Reef Alruqaie, Fernando Altermatt, Bruno Luís de Castro Araujo, Genaro Arbesú, Hanna Artsi, Caterina Aurilio, Omer Hilmi Ayanoglu, Alessandro Bacuzzi, Harris Baig, Yolanda Baird, Konstantin Balonov, Jaume Balust, Samantha Banks, Xiaodong Bao, Mélanie Baumgartner, Isabel Belda Tortosa, Alice Bergamaschi, Lars Bergmann, Luca Bigatello, Elena Biosca Pérez, Katja Birr, Thomas Bluth, Ilona Bobek, Elird Bojaxhi, Chiara Bonenti, Iwona Bonney, Elke M. E. Bos, Sara Bowman, Leandro Gobbo Braz, Elisa Brugnoni, Sorin J. Brull, Iole Brunetti, Andrea Bruni, Shonie L. Buenvenida, Cornelius Johannes Busch, Giovanni Camerini, Jaume C. Canet, Beatrice Capatti, Javiera Carmona, Jaime Carungcong, Marta Carvalho, Anat Cattan, Carla Cavaleiro, Davide Chiumello, Stefano Ciardo, Mark Coburn, Umberto Colella, Victor Contreras, Pelin Corman Dincer, Elizabeth Cotter, Marcia Crovetto, William Darrah, Simon Davies, Luc de Baerdemaeker, Stefan De Hert, Enrique Del Cojo Peces, Ellise Delphin, John Diaper, Paulo do Nascimento Junior, Valerio Donatiello, Jing Dong, Maria do Socorro Dourado, Alexander Dullenkopf, Felix Ebner, Hamed Elgendy, Christoph Ellenberger, Dilek Erdoğan Arı, Thomas Ermert, Fadi Farah, Ana Fernandez-Bustamante, Cristina Ferreira, Marco Fiore, Ana Fonte, Christina Fortià Palahí, Andrea Galimberti, Marcelo Gama de Abreu, Najia Garofano, Luca Gregorio Giaccari, Fernando Gilsanz, Felix Girrbach, Luca Gobbi, Marc Bernard Godfried, Nicolai Goettel, Peter A. Goldstein, Or Goren, Andrew Gorlin, Manuel Granell Gil, Angelo Gratarola, Juan Graterol, Cesare Gregoretti, Pierre Guyon, Kevin Haire, Philippe Harou, Göran Hedenstierna, Antonia Helf, Sabrine N. T. Hemmes, Gunther Hempel, María José Hernández Cádiz, Björn Heyse, Michael Hiesmayr, Markus W. Hollmann, Ivan Huercio, Jasmina Ilievska, Samir Jaber, Lien Jakus, Vijay Jeganath, Yvonne Jelting, Minoa Jung, Barbara Kabon, Aalok Kacha, Maja Karaman Ilić, Arunthevaraja Karuppiah, Ayse Duygu Kavas, Gleicy Keli Barcelos, Todd A. Kellogg, Johann Kemper, Romain Kerbrat, Suraya Khodr, Peter Kienbaum, Bunyamin Kir, Thomas Kiss, Selin Kivrak, Vlasta Klarić, Thea Koch, Ceren Köksal, Ana Kowark, Peter Kranke, Bahar Kuvaki, Biljana Kuzmanovska, John Laffey, Mirko Lange, Marília Freitas de Lemos, Marc-Joseph Licker, Manuel López-Baamonde, Antonio López-Hernández, Mercedes Lopez-Martinez, Stéphane Luise, Mark MacGregor, Danielle Magalhães, Julien Maillard, Patrizia Malerbi, Natesan Manimekalai, Michael Margarson, Klaus Markstaller, Archer K. Martin, David P. Martin, Yvette N. Martin, Julia Martínez-Ocon, Ignacio Martin-Loeches, Emilio Maseda, Idit Matot, Niamh McAuliffe, Travis J. McKenzie, Paulina Medina, Melanie Meersch, Angelika Menzen, Els Mertens, Bernd Meurer, Tanja Meyer-Treschan, Changhong Miao, Camilla Micalizzi, Morena Milić, Gary H. Mills, Norma Sueli Pinheiro Módolo, Pierre Moine, Patrick Mölders, Ana Montero-Feijoo, Enrique Moret, Jan Paul Mulier, Markus K. Muller, Zoe Murphy, Pramod Nalwaya, Filip Naumovski, Paolo Navalesi, Lais Helena Navarro e Lima, Višnja Nesek Adam, Claudia Neumann, Christopher Newell, Zoulfira Nisnevitch, Junaid Nizamuddin, Cecilia Novazzi, Michael O’Connor, Günther Oprea, Mukadder Orhan Sungur, Şule Özbilgin, Maria Caterina Pace, Marcos Pacheco, Balaji Packianathaswamy, Estefania Palma Gonzalez, Fotios Papaspyros, Sebastián Paredes, Maria Beatrice Passavanti, Juan Cristobal Pedemonte, Paolo Pelosi, Sanja Peremin, Christoph Philipsenburg, Daniela Pinho, Silvia Pinho, Linda M. Posthuma, Vincenzo Pota, Benedikt Preckel, Paolo Priani, Christian Putensen, Mohamed Aymen Rached, Aleksandar Radoeshki, Riccardo Ragazzi, Tamilselvan Rajamanickam, Arthi Rajamohan, Harish Ramakrishna, Desikan Rangarajan, Christian Reiterer, J. Ross Renew, Thomas Reynaud, Rhidian Rhys, Eva Rivas, Luisa Robitzky, Rolf Rossaint, Francesca Rubulotta, Humberto S. Machado, Catarina S. Nunes, Giovanni Sabbatini, Jon D. Samuels, Josep Martí Sanahuja, Pasquale Sansone, Alice Santos, Mohamed Sayedalahl, Maximilian S. Schaefer, Martin Scharffenberg, Eduardo Schiffer, Nadja Schliewe, Jochen Schmitt, Raoul Schorer, Marcus J. Schultz, Roman Schumann, Gabriele Selmo, Mar Sendra, Mert Senturk, Ary Serpa Neto, Paolo Severgnini, Kate Shaw, Mirjana Shosholcheva, Abdulrazak Sibai, Philipp Simon, Francesca Simonassi, Claudia Sinno, Nukhet Sivrikoz, Vasiliki Skandalou, Neil Smith, Maria Soares, Tania Socorro Artiles, Diogo Sousa Castro, Miguel Sousa, Savino Spadaro, Juraj Sprung, Emmanouil Stamatakis, Luzius A. Steiner, Andrea Stevenazzi, Alejandro Suarez-de-la-Rica, Mélanie Suppan, Robert Teichmann, José Maria Tena Guerrero, Bram Thiel, Raquel Tolós, Gulbin Tore Altun, Michelle Tucci, Zachary A. Turnbull, Žana Turudić, Matthias Unterberg, Jurgen Van Limmen, Yves Van Nieuwenhove, Julia Van Waesberghe, Marcos Francisco Vidal Melo, Bibiana Vitković, Luigi Vivona, Marcela Vizcaychipi, Carlo Alberto Volta, Anne Weber, Toby N. Weingarten, Jakob Wittenstein, Hermann Wrigge, Piet Wyffels, Julio Yagüe, David Yates, Ayşen Yavru, Lilach Zac, and Jing Zhong
